# Supplementary material for: COZOID: contact zone identifier for visual analysis of protein-protein interactions
Source: BMC Bioinformatics. 2018 Apr 6;19:125. doi: 10.1186/s12859-018-2113-6 (PMC5889581; doi:10.1186/s12859-018-2113-6)
Supplement: Supplementary file 2 — Software build. Executable binary file of the software tool. (ZIP 118,784 kb) [file 12859_2018_2113_MOESM2_ESM.zip › cozoid/platform/docs/testng-6.5.1-javadoc.zip!/org/testng/annotations/package-frame.html]

org.testng.annotations (TestNG)


org.testng.annotations

Interfaces 
  
*IAnnotation*
  
*IConfigurationAnnotation*
  
*IDataProviderAnnotation*
  
*IExpectedExceptionsAnnotation*
  
*IFactoryAnnotation*
  
*IObjectFactoryAnnotation*
  
*IParameterizable*
  
*IParametersAnnotation*
  
*ITestAnnotation*
  
*ITestOrConfiguration*

Annotation Types 
  
AfterClass
  
AfterGroups
  
AfterMethod
  
AfterSuite
  
AfterTest
  
BeforeClass
  
BeforeGroups
  
BeforeMethod
  
BeforeSuite
  
BeforeTest
  
Configuration
  
DataProvider
  
ExpectedExceptions
  
Factory
  
Guice
  
Listeners
  
NoInjection
  
ObjectFactory
  
Optional
  
Parameters
  
Test
  
TestInstance
